# Application of role reversal and standardized patient simulation (SPS) in the training of new nurses

**DOI:** 10.1186/s12909-023-04294-1

**Published:** 2023-05-24

**Authors:** Yingmin Liu, Dandan Qie, Mengmeng Wang, Yuyuan Li, Dongmei Guo, Ximin Chen, Li Li, Honglian Yu, Jingjing Wang

**Affiliations:** Department of Nursing, The No.2 Hospital of Baoding, No. 338 Dongfeng West Road, Jingxiu District, Baoding, 071051 Hebei Province China

**Keywords:** Role reversal, Standardized vertebral patients, Hiring a nurse, Training work, Training satisfaction

## Abstract

**Purpose:**

To explore the effect of role reversal and standardized patient simulation on the training of new nurses.

**Method:**

This study was conducted in a territory hospital in China between August 2021 and August 2022. The selected staff were all newly recruited and trained nurses, with a total of 58 cases. This study is a randomised controlled trial. The selected nurses were randomly divided into two groups. One group of 29 nurses (the control group) received routine training and assessment; the other group (the experimental group) was given role reversal combined with a standardized vertebral patient training examination. The implementation effects of different training and assessment methods were compared and analysed.

**Results:**

Before the training, the core competence scores of nurses in the two groups were lower, and there was no significant data difference (*P* > 0.05). After training, the core competence scores of nurses were improved, and the score of nurses in the experimental group was 165.49 ± 22.34. The difference was statistically significant when compared with the score of nurses in the control group (*P* < 0.05), indicating that nurses in the experimental group had better abilities. At the same time, the satisfaction of the two groups of nurses with the training was 96.55% (experimental group) and 75.86% (control group), and the difference in data was significant (*P* < 0.05). The satisfaction of the experimental group of nurses was higher, and the training effect was better.

**Conclusion:**

In the training of new nurses, the combined application of role interchange and standardized patient training and assessment methods has significant effects, which can improve the core competency of nurses and improve the training satisfaction of nurses, which is significant.

## Introduction

The pre-employment training methods for new nurses in clinical work are relatively straightforward, and the training results are uneven [1]. As a new pre-employment training method, role reversal allows nurses to take on the roles of patients. Using this method, they put themselves in a patient’s position and realistically simulate the process of a medical visit [2]. Role reversal can significantly improve training efficiency, but there are few relevant studies. This study found that the application of role reversal and standardized patient training and assessment methods in the training of new nurses has significant effects, which can considerably improve the core competency and training satisfaction of nurses. It provides new guidance and suggestions for the pre-job training of new nurses in the future.

In 2016, China’s National Health Commission organised and formulated the *Training Outline for New Nurses* in order to guide the training of new nurses in medical institutions across China and improve the quality of training so as to improve the nursing service ability of nurses and meet the health needs of the people [3]. The responsibilities of nurses in hospital services are not limited to administering first-aid and emergency treatment. Many kinds of adverse situations occur regularly in the hospital setting, so it is particularly important to improve the nursing abilities of nurses [4]. Newly recruited nurses are mainly nurses who entered clinical nursing after graduating from colleges and universities. Hospitals need to train these newly recruited nurses according to their previous work experience to improve their ability to handle emergencies. In the process of standardising training for nurses, different training and assessment methods have been found to have differing effects on improving the ability of new nurses [5]. This set of teaching and training methods mainly include role conversion and standardized patient simulation (SPS), focusing on the improvement of clinical nurses’ abilities, combined with the teaching model of standardized patients participating in the demonstration of clinical scenarios, to provide new nurses with more vivid and authentic clinical comprehensive ability training and assessment. These training methods attach great importance to the cultivation of nursing quality, demonstrate humanistic care, and fully reflect the integrity and humanity of nursing work. They effectively embody the perfect combination of clinical role conversion and standardized patient training [6]. Based on this information, this study examined nurses in our hospital as the research subjects, combined the role reversal and SPS training assessment methods, and compared and analysed their clinical implementation effects.

## Materials and methods

### Research design and methods

This research experiment was conducted in a territory hospital from August 2021 to August 2022. A total of 58 newly recruited nurses were selected as the research subjects. This study was mainly a comparative experiment. Nurses were randomly divided into two groups, the experimental group and the control group, according to the random number table method. Nurses in the control group received routine training methods, and the specific steps were as follows. Nurses were provided with centralised training that explained nursing knowledge, informed them of specific emergency treatment methods, and made them aware of relevant regulations to ensure that they could complete the tasks required by various departments. After the training was completed, assessment of the staff was carried out. The traditional paper examination method and practical training method were mainly used to comprehensively assess the scores. The nurses who passed the examination were then allowed to work.

Nurses in the experimental group were trained using role reversal combined with SPS, and the specific steps were as follows. The knowledge training method was the same as the control group, and the assessment method was carried out in the form of role reversal combined with standardized vertebral patients. In the specific implementation process, the new nurses were assessed, and the assessment methods of role reversal and SPS were introduced into the assessment items. Before the assessment, it was necessary to guide new nurses in how to properly simulate standardized patients. The nurses needed to understand and master the patients’ medical records and focus on the patients’ nursing needs. Nurses that were playing the role of standardized patients needed to utilise their imaginations to simulate not only the disease symptoms but also the emotional and mental states of such patients, including fear and anxiety. For example, patients with bronchiectasis would be afraid that they would die because they were coughing up blood; patients undergoing an operation for acute appendicitis would be both worried before the operation and painful after it, etc. In order to better simulate standardized patients, new nurses were divided into two groups. One group consisted of nurses under assessment; the other consisted of nurses playing the role of standardized patients. The assessment of the two groups of new nurses was then done. Assessment mainly included the thoroughness with which standardized patients were handled, including the nurses’ attitudes and competency with technical skills. After completing a round of tests, the two groups were switched, with one person playing the role of the standardized patient and the other performing the same nursing tasks. After the assessment, the assessors encouraged the new nurses to express their feelings and opinions about the experience, including what they found clarifying about the process and how they thought it would help them pay better attention and do better nursing work in the future. To evaluate the results, in addition to the conventional assessment of the teacher’s score, this assessment also needed to introduce the SPS score of the examiners, including the nurses’ actions and sentiments, the summary, etc., to make a comprehensive evaluation, so as to ensure the assessment’s accuracy.

### Data analysis

The double-recording method was used, and EpiData 3.0 software was used to input data. After the input was completed, it was converted into an SPSS data set. SPSS statistical software was used for the descriptive statistical analysis of general data. Frequency, composition ratio, mean standard deviation (x ± s), the coefficient of variation, and the measurement data that conformed to the normal distribution after the test was described. The comparison between the groups was performed using two independent sample t-tests, and the count data comparison was performed using the chi-squared test. The difference was statistically significant at *P* < 0.050.

The following conditions were necessary for inclusion: all participating personnel were new graduates, and all participating personnel agreed with the research process and agreed to cooperate with the experiment. This study was approved by the ethics committee of our hospital.

Exclusion criteria were as follows: poor compliance of personnel, inability of personnel to cooperate with the experiment, and status of personnel as experienced employees instead of new graduates.

### Index of observation

To evaluate the nursing ability of the two groups of nurses, the Chinese Nurse Core Competence Scale [7] was applied, which evaluated 58 items and 7 dimensions involving clinical nursing ability, interpersonal ability, critical thinking, leadership, education/consulting ability, etc. The scale score ranged from 0 to 220, and the higher the score, the stronger the core competency of the nurse and the better the effects of the training. The Cronbach’s coefficient of the questionnaire was greater than 0.7, indicating it could be used to produce reliable, valid data.

The training satisfaction of the two groups of nurses was investigated using a satisfaction questionnaire made by our hospital. The full score of this questionnaire was 100, and the higher the score, the better the nurse’s satisfaction. Based on their scores, they were categorised as very satisfied (above 90), satisfied (70–90) and dissatisfied (below 70). The total satisfaction score was the sum of very satisfied and satisfied respondents.

## Results

### General demographic information

In the experimental group, there were 29 subjects, including 3 males and 26 females. The oldest was 25 years old, the youngest was 23 years old, and the mean age was 24.33 ± 0.34 years old. There were 15 patients with bachelor’s degrees and 14 patients with college degrees. In the control group, there were 2 males and 27 females, aged between 23 and 25 years old, and the corresponding mean age was 24.34 ± 0.23 years old. Among them, there were 16 participants with bachelor’s degrees and 13 participants with college degrees.

### Core competence scale scores of nurses in two groups

Before the training, the core competence scores of nurses in the two groups were lower, and there was no significant data difference (*P* > 0.05). After training, the core competence scores of nurses improved, and the training degree of the experimental group was higher. The data difference was statistically significant when compared with the score of the control group (*P* < 0.05). The ability of nurses in the experimental group was better, as shown in Table 1 below.

**Table 1:** The scores of core nursing competence scale were compared between the two groups (x ± S)

group	Before training (scores)	After training (scores)
experimental group (*n* = 29)	110.54 ± 23.34	165.49 ± 22.34
control group (*n* = 29)	111.52 ± 23.11	148.43 ± 23.20
*t*	0.161	2.852
*P*	0.873	0.006

### Training satisfaction survey of nurses

We investigated the training satisfaction of nurses through the satisfaction questionnaire. The satisfaction of the experimental and control groups with training was 96.55% and 75.86%, respectively, and the difference in data was significant, expressed as *P* < 0.05. The satisfaction of the experimental group was higher, as shown in Table 2.

**Table 2:** The nurses’ satisfaction with the training was compared between the two groups [n (%)]

group	great satisfaction	satisfaction	dissatisfaction	Satisfaction value
experimental group (*n* = 29)	20 (68.96)	8 (27.59)	1 (3.45)	28 (96.55)
control group (*n* = 29)	12 (41.38)	10 (34.48)	7 (24.14)	22 (75.86)
χ^2^				5.220
*P*				0.022

## Discussion

In the clinical development of China, most of the newly recruited nurses are fresh graduates who do not understand actual clinical development. They lack emergency response ability and nursing ability and are prone to overstrain, which can easily lead to a variety of adverse events in nursing work [8]. In order to avoid the occurrence of this kind of situation, the health department requires the newly recruited nurses to take up their posts only after passing the hospital training and examination, so as to ensure effective clinical nursing services. However, in the development of clinical practices in the past, traditional teaching methods were usually adopted. These methods are one-way, and it was difficult to give full play to the subjective initiative of students in learning [9]. They only provided conclusive knowledge for new nurses, which was not beneficial to improve nurses’ autonomous learning ability [10]. With role transformation and the standard of the vertebra patient training method, nurses are required to use multiple aspects of their abilities, such as sight, hearing, touch, and motion coordination. The effects on new nurses are positive due to active thinking, which effectively combines abstract thinking with image thinking, and experience sharing. This inspires reasoning and helps nurses better grasp the nursing method and process [11]. The results of relevant research data show that the research into and reform of the content and training methods of pre-job instruction have positive significance for the role transformation of nurses [12]. Therefore, the combined application of role transformation and the SPS method in the training of new nurses can improve the effectiveness of training and ensure the comprehensive ability of nurses. Role-playing can help nurses enhance patients’ comfort with hospitalization and help new nurses standardise service behaviours. This process is a personal experience, in which nurses can fully feel the needs of patients. For example, in the process of oral care, the new nurses will feel the discomfort caused by too much or too little water on the cotton balls when they are standardized patients. This feeling will make the new nurses realise the importance of their work. Nurses will then be more likely to avoid the occurrence of this problem in their future work by providing reasonable control of cotton ball moisture. This will improve the quality of nursing and ensure their nursing care has positive effects.

In role reversal and standardized patient training, nurses can experience the feelings of patients during hospitalization. In order to better simulate the actual situations of patients, nurses need to not only understand a patient’s condition but also better understand a patient’s feelings. This process helps nurses give better nursing service experiences to patients after admission. The characteristic of role reversal combined with the SPS method is personal experience, which includes the personal experience of nurses and the personal experience of ‘heart’. Personal experience can better touch people’s hearts to generate emotional resonance, which then improves the quality of care [13]. In this process, role-playing can deepen the influence of nurses on ‘patients’ in the scene through situational experience, so that nurses can understand that their words and actions will have an impact on patients, which will help them better regulate their own behaviour and provide patients with needed nursing services. The whole simulation exercise process can help new nurses understand the necessity of mastering instrument skills, humanistic care, communication skills, and normal processes, as well as improve their consciousness while strengthening the practice, so as to standardise their own service behaviour. This method can help nurses practise actual nursing work, so as to avoid unproficiency after entry, which can lead to patients’ over-tension. This has positive significance for improving nurses’ core operational ability.

The application of role reversal and SPS can help nurses experience the feelings of inpatients and promote the consideration of new nurses. Relevant research data show that thinking about the needs of patients from the perspective of nurses often ignores the feelings and needs of patients because of a strong professional perspective [3]. Some researchers have also expressed that nurses can change their identity through role reversal when carrying out role-play work and consider nursing issues from the perspective of patients, which has positive significance for cultivating nurses’ patient-centred service attitude. In the process of this study, nurses experienced patients’ admission psychology, fear psychology, and helplessness from the perspective of role reversal as standardized patients. This helped them understand patients’ needs more effectively, which caused them to provide better service quality according to patients’ needs. For example, nurses can actively communicate with patients after understanding their nervous, unfamiliar, and worried psychological state at admission in order to better introduce the hospital environment to patients and relieve their nervousness, which ensures better nursing services.

At the same time, role reversal and standardized patient training can guide new nurses to implement humanistic care. In the process of clinical nursing work, it is necessary to reflect on the people-oriented concept of nursing so that patients’ psychological, physiological, and social aspects are satisfied, so that patients are in a relatively comfortable state [4]. In this process, nurses are required to respect and understand patients, integrate humanistic care throughout their whole nursing work, and give patients care and warmth with friendly smiling faces, encouraging eyes, and comforting actions, so as to improve the quality of nursing service of new nurses [5].

In addition, role reversal combined with standardized patient training can improve the ability of nurses to solve nursing problems. The traditional admission nursing method usually explains the admission nursing of general patients and emergency patients step by step. The teaching method of the instructor in this method is relatively boring, which can cause the nurses to have a low degree of knowledge retention after class, resulting in the effect of poor knowledge impartation. After adopting the method of role transformation, nurses need to perform personalised nursing role-playing for patients according to different patients and different diseases, which has positive significance for cultivating nurses’ flexible problem-solving ability [14]. At the same time, research shows that people build and consolidate knowledge in the process of metacognition. This attitude usually affects conceptual construction; therefore, nurses in the process of learning are more willing to study different types of knowledge. This method is thus more conducive to the nurse mastering good methods and problem-solving abilities [8]. The method of role experience can increase the nurses’ interest in the learning process, which can create a good learning atmosphere and further change the learning attitude of new nurses to admission nursing, so as to promote the transformation of theoretical nursing knowledge to practical skills. In the process of training, nurses acting as patients in a variety of emergency situations leads to greater empathy, which is also an important factor in training the ability of new nurses. This also enhances their ability to deal with clinic strain and is an important basis for improving the nursing ability of new nurses.

In this study, the role transformation and SPS training assessment methods were combined, and the results showed that after training, nurses in the experimental group had a higher score of core nursing competence. Nurses in this group also had higher satisfaction with their training, and all the data were significantly different from those in the control group using the traditional training method (*P* < 0.05). The results of this study are similar to the results of Britt Cole’s study [15], which proves that the use of role transformation and the standardized patient method have positive significance in improving training effectiveness and enhancing the ability of nurses and confirms the above research theories. However, this study was only evaluated in the hospital setting and still needs to be applied to clinical work. Through clinical practice, patients can make the final evaluation on the working ability of nurses.

## Conclusion

In summary, the application of role conversion and standardized patient training methods to train new nurses can improve the core competence and training satisfaction of nurses, which has high clinical application value and can be promoted in clinical practice. However, the sample size of the research subjects in this experiment was small, and it is recommended to expand the sample size in the future to further verify the training effect.

## Data Availability

All data generated or analyzed during this study are included in this published article.
